# Defining consensus opinion to develop randomised controlled trials in rare diseases using Bayesian design: An example of a proposed trial of adalimumab versus pamidronate for children with CNO/CRMO

**DOI:** 10.1371/journal.pone.0215739

**Published:** 2019-06-05

**Authors:** A. V. Ramanan, L. V. Hampson, H Lythgoe, A. P. Jones, B Hardwick, H Hind, B Jacobs, D Vasileiou, I Wadsworth, N Ambrose, J Davidson, P. J. Ferguson, T Herlin, A Kavirayani, O. G. Killeen, S Compeyrot-Lacassagne, R. M. Laxer, M Roderick, J. F. Swart, C. M. Hedrich, M. W. Beresford

**Affiliations:** 1 Paediatric Rheumatology, Bristol Royal Hospital for Children, Bristol and Bristol Medical School, University of Bristol, Bristol, United Kingdom; 2 Statistical Methodology and Consulting, Novartis Pharma AG, Basel, Switzerland; 3 Department of Women's & Children's Health, Institute of Translational Medicine, University of Liverpool, Liverpool, United Kingdom; 4 Department of Paediatric Rheumatology, Alder Hey Children’s NHS Foundation Trust, Liverpool, United Kingdom; 5 Clinical Trials Research Centre, Department of Biostatistics, University of Liverpool, Liverpool, United Kingdom; 6 Paediatrics, Royal National Orthopaedic Hospital, London, United Kingdom; 7 Department of Mathematics and Statistics, Fylde College, Lancaster University, Lancaster, United Kingdom; 8 Rheumatology, University College Hospital, London, United Kingdom; 9 Paediatric Rheumatology, Royal Hospital for Children, Glasgow, United Kingdom; 10 Stead Family Department of Pediatrics, University of Iowa Carver College of Medicine, Iowa City, IA, United States of America; 11 Department of Paediatrics, Aarhus University, Aarhus, Denmark; 12 Paediatric Rheumatology, Oxford University Hospitals NHS Foundation Trust, Oxford, United Kingdom; 13 National Centre for Paediatric Rheumatology, Our Lady’s Children Hospital, Crumlin, Dublin, Ireland; 14 Rheumatology, Great Ormond Street Hospital for Children NHS Foundation Trust, London, United Kingdom; 15 Department of Paediatrics, University of Toronto, The Hospital for Sick Children, Toronto, Canada; 16 Paediatric Rheumatology, University Medical Centre Utrecht, Utrecht, Netherlands; University of Ottawa, CANADA

## Abstract

**Introduction:**

Chronic nonbacterial osteomyelitis (CNO) is a rare autoinflammatory bone disorder primarily affecting children and adolescents. It can lead to chronic pain, bony deformities and fractures. The pathophysiology of CNO is incompletely understood. Scientific evidence suggests dysregulated expression of pro- and anti-inflammatory cytokines to be centrally involved. Currently, treatment is largely based on retrospective observational studies and expert opinion. Treatment usually includes nonsteroidal anti-inflammatory drugs and/or glucocorticoids, followed by a range of drugs in unresponsive cases. While randomised clinical trials are lacking, retrospective and prospective non-controlled studies suggest effectiveness of TNF inhibitors and bisphosphonates. The objective of the Bayesian consensus meeting was to quantify prior expert opinion.

**Methods:**

Twelve international CNO experts were randomly chosen to be invited to a Bayesian prior elicitation meeting.

**Results:**

Results showed that a typical new patient treated with pamidronate would have an 84% chance of improvement in their pain score relative to baseline at 26 weeks and an 83% chance on adalimumab. Experts thought there was a 50% chance that a new typical patient would record a pain score of 28mm (pamidronate) to 30mm (adalimumab) or better at 26 weeks. There was a modest trend in prior opinion to indicate an advantage of pamidronate vs adalimumab, with a 68% prior chance that pamidronate is superior to adalimumab by some margin. However, it is clear that there is considerable uncertainty about the precise relative merits of the two treatments.

**Conclusions:**

The rarity of CNO leads to challenges in conducting randomised controlled trials with sufficient power to provide a definitive outcome. We address this using a Bayesian design, and here describe the process and outcome of the elicitation exercise to establish expert prior opinion. This opinion will be tested in the planned prospective CNO study. The process for establishing expert consensus opinion in CNO will be helpful for developing studies in other rare paediatric diseases.

## Introduction

Chronic nonbacterial osteomyelitis (CNO) is a rare bone disorder producing sterile inflammatory lesions. While some patients show timely limited monofocal disease, others will develop chronically active or recurrent courses with multifocal bone involvement, which is then referred to as chronic recurrent multi-focal osteomyelitis (CRMO) [1]. Primarily affecting children and adolescents, CNO/CRMO is characterized by the insidious onset of bone pain that may be severe and disabling, potentially leading to permanent damage [2].

The molecular pathophysiology is poorly understood. Currently, CNO is widely considered to be an autoinflammatory disorder characterised by imbalanced expression of pro- and anti-inflammatory cytokines [3–7]. Currently available treatment is based on case reports, retrospective or uncontrolled prospective case collections and expert opinion. Treatment strategies for children with CNO/CRMO vary widely. Initially, treatment often includes nonsteroidal anti-inflammatory drugs (NSAIDS) and/or glucocorticoids. Where these treatments are ineffective, a range of drugs have been tried including: bisphosphonates, sulfasalazine, methotrexate and anti-TNF agents [3–15]. Among these options, the bisphosphonate pamidronate and TNF inhibitors are considered most effective [3–15].

Pro-inflammatory TNF-α is involved in the differentiation and activation of osteoclasts, which potentially centrally contributes to bone inflammation in CNO/CRMO. Thus, TNF inhibition may (at least partially) correct cytokine imbalance in CNO. Indeed, several reports indicate successful use of anti-TNF treatment in the management of CNO patients, including patients who were refractory to pamidronate [8, 16].

The mechanism of action of bisphosphonates is uncertain [16]. The current hypothesis is that bisphosphonates inhibit pathologically activated osteoclasts and may (partially) correct imbalanced cytokine expression in CNO/CRMO [16].

Based on aforementioned retrospective reports, observational studies and expert opinion, consensus treatment plans were developed by the North American Childhood Arthritis & Rheumatology Research Alliance (CARRA) group [17]. However, to date, there have been no published randomised controlled trials (RCTs) in CNO/CRMO. Indeed, phase III RCTs in CNO/CRMO are unlikely to be feasible at this point in time, since patients are rare, preliminary reports suggest efficacy of anti-inflammatory treatment, and the absence of financial incentives to industry. To generate urgently needed reliable evidence for safety and efficacy of anti-inflammatory treatment in CNO, we propose that a Phase II study, randomising CNO/CRMO patients aged 6 to 18 years to either pamidronate or adalimumab treatment. On the basis of feasibility analyses, it is expected that approximately 40 children and young adults with CNO/CRMO could be recruited across a UK network of 12 centres in two years. It is likely that on the basis of such a sample size, a conventional hypothesis testing trial would have low power to reliably detect a difference between treatments. However, this sample size is large enough to be clinically relevant. With this in mind, we propose a Phase II trial to be performed using Bayesian trial design [18].

Bayesian design is an innovative approach to clinical studies in small, but potentially diverse patient populations [18]. There is growing interest in such approaches to facilitate the design and interpretation of trials, as evidenced by the recent Food and Drug Administration (FDA) workshop on this topic [19]. In the rare disease setting, by the time an RCT is planned, off-label prescribing of the medicine of interest may already be underway. The Bayesian approach can be used to formally augment data from a new RCT with clinicians’ prior understanding of each medicine’s efficacy and safety. This understanding may be informed by several sources, including the clinicians’ own prescribing experience, as well as existing published evidence. An essential first step in a Bayesian trials is to carefully record available knowledge before the new RCT begins. Many approaches have been proposed for eliciting experts’ individual opinions and for aggregating them to summarise the position of a group [20–22]. We followed the approach adopted for the MYPAN trial in polyarteritis nodosa [23, 24] and convened a face-to-face meeting of Paediatric Rheumatologists experienced in treating CNO/CRMO and recognized as national and/or international experts. We used behavioural aggregation to establish consensus prior distributions that will underpin a future Phase II trial in CNO/CRMO.

## Methods

### Establishing a group of experts to determine consensus prior opinion

A one-day consensus meeting was organised to bring together an international group of CNO/CRMO experts from Europe and North America. We defined an expert as a clinician who: a) had a documented interest in CNO/CRMO and would consider themselves to be a local expert; b) was a consultant-grade clinician and specialist in paediatric rheumatology or orthopaedics; c) had treated at least 10 cases of CNO/CRMO in the last three years. Available funding permitted the attendance of thirteen experts at the consensus meeting. Attendance by two internationally recognised leading CNO/CRMO researchers based in North America (PF, RL) was considered essential to ensure that the wider research community would adopt the eventual outcome of the consensus meeting. Within these constraints, we set out to identify six further clinicians from the UK and Ireland, and four clinicians from the rest of Europe who satisfied our expert criteria and could attend in-person a one-day meeting in London, UK on 1^st^ July 2016.

To identify potential experts, targeted enquiries were sent to research groups known to be highly active in CNO/CRMO research which had published in high impact peer-reviewed medical journals (PubMed journals with Impact Factor >/ = 4.0) in the last five years (2011–2016). Such groups were invited to nominate a member to be considered for the meeting. In addition, e-mails were circulated to members of the British Society of Paediatric and Adolescent Rheumatology (BSPAR; http://www.rheumatology.org.uk/bspar/) and the Paediatric Rheumatology European Society (PRES; http://www.pres.eu) inviting expressions of interest. All respondents were asked to complete an electronic survey confirming that they met the stated criteria for meeting participation.

*A priori* it was agreed that meeting participants would be randomly selected from the group of eligible experts identified through the process described above. Selection followed a pre-specified protocol which stipulated that six experts, representing different centres, would be randomly selected from the UK and Ireland, while four experts from different countries would be randomly selected from the rest of Europe. All participants volunteered to take part in the prior elicitation meeting as experts. No patients were involved in the meeting. Ethics approval was therefore not required.

### Process of establishing consensus prior opinion

The meeting took place in London on 1^st^ July 2016 and proceeded according to the agenda listed in Table 1, which itself emulates the agenda of the prior elicitation meeting performed for the MYPAN trial in polyarteritis nodosa [24]. Prior to the elicitation meeting, experts were sent preparatory reading material which comprised a summary of the key elements that would feature in the protocol of a future CRMO trial. The meeting then began with a recap of this material and an overview of the diagnosis of CRMO and existing information supporting the use of pamidronate and adalimumab in the management of CRMO (drawn from two retrospective reviews of: a) 11 children with a CNO diagnosis who had received pamidronate therapy [25]; b) a US cohort of 70 children diagnosed with CNO, of whom 11 were treated with anti-TNF agents [26]). Other training sessions during the elicitation meeting covered the clinical motivation for a future CRMO trial and its potential design. An introduction to the Bayesian approach and a practical, interactive, example of the prior elicitation process was also provided by a statistician with experience of Bayesian clinical trials.

**Table 1:** Activities comprising the consensus meeting and the time dedicated to each.

Time allocation (minutes)	Activity
• 30	**Overview** • Introduction to the planned CRMO trial and the scope of the elicitation meeting
• 30 • 30	**Training on Bayesian statistics** • Seminar introducing the Bayesian approach and how it can be used to represent prior opinion. • Practical providing experts with an opportunity to describe their own uncertainty in the context of a non-substantive example.
• 30	**Clinical overview of existing CRMO treatment options** • Review of current treatment options for CRMO and what is known about them
• 15	**Rationale for the Bayesian CRMO trial design** • Discuss rationale for adopting Bayesian design for CRMO trial
• 90 • 30 • 60 • 30	**Formal elicitation exercise** • Each expert completes an individual elicitation questionnaire, then meets with a statistical facilitator to visualise the prior corresponding to their stated answers. This process is iterated until the expert is content that the prior reflects their underlying beliefs. • Present to the group all individuals’ answers to the ten primary elicitation questions (QP1-QP5 and QA1-QA5 listed in S1 Appendix). • Convene structured discussions to identify consensus answers to QP1-QP5 and QA1-QA5. If a consensus cannot be reached, characterise the conflicting opinions of the distinct expert subgroups. • Present visualisations and summaries of the prior distributions corresponding to consensus answers (if these can be established). Establish the validity of these priors as representing the consensus prior opinion of the group.

The afternoon was devoted to the formal elicitation process, structured so that the opinions of individuals were established before attempting to reach a consensus [27]. We adopted this structure to reduce the risk of experts being unduly influenced by overconfident group members or those with strong personalities. Four facilitators with statistical training were on hand throughout to facilitate (LVH, APJ, DV and IW). Each expert was first asked to complete a structured questionnaire before having a one-to-one meeting with a facilitator. The facilitator took the expert’s answers to ten questions and fed back visualisations and summaries of the consequences of the stated beliefs: the expert was permitted to refine his/her initial answers until priors with face validity could be obtained. Once each expert’s individual opinions had been finalised, these were displayed to the group and each expert was invited to comment on his or her answers. Structured discussions ensued, moderated by the two chief clinical investigators (AVR, MWB) and a statistical facilitator (LVH). During these discussions, experts were permitted to update their answers to the 10 key elicitation questions, although only one expert took advantage of this opportunity. Finally, we used the approach outlined in section ‘Establishing consensus expert opinion’ to identify consensus answers to the ten primary elicitation questions, which the majority could adopt as adequate reflections of their prior beliefs. These consensus opinions, and their consequences, are detailed below.

## Statistical approach for establishing Bayesian prior distributions

### Defining the quantities to be elicited

The primary endpoint of a future Phase II CNO/CRMO trial will be change in pain score from baseline at 26 weeks, measuring pain scores on a 100 mm visual analogue scale (VAS). We assumed that patient outcomes will follow a Gaussian ‘bell-shaped’ distribution, with a common variance across treatment arms. This is a pragmatic model since it does not adjust for baseline scores. Neither does it formally account for the fact that pain scores must lie between 0 and 100 mm, although it should be reasonably accurate as long as pain scores do not tend to lie too close to either boundary. Denoting by μ_P_ the average outcome that would be observed across a large number of patients randomised to pamidronate, we can express the corresponding average outcome on adalimumab as μ_P_ + δ. Thus δ, which is the difference between average outcomes on adalimumab and pamidronate, is the ‘treatment effect’ of interest. Since a negative change in pain score implies that a patient’s symptoms have improved from baseline, a negative value for δ is consistent with adalimumab having superior efficacy relative to pamidronate, and vice versa. We refer to the common variance of patient outcomes as 1/τ.

Together, μ_P_, δ and τ define the likelihood of observing any potential dataset from the future CRMO trial. The objective of the Bayesian consensus meeting was to quantify prior opinion on these three quantities. Once data from a future CRMO trial become available, Bayes Theorem will be used to update this prior opinion to obtain posterior distributions representing the totality of what has been learnt about the relative merits of pamidronate and adalimumab.

### Statistical model for expert prior opinion

We adopted a ‘conjugate’ model for prior opinion on the three quantities (μ_P_, δ and τ) defined above, so-called because posterior distributions incorporating trial data will be of the same statistical form as the prior distributions. Specifically, we assumed that prior opinion on τ could be modelled as a gamma distribution with shape and rate parameters a_0_ and b_0_, respectively. Furthermore, we assumed that if the true value of τ were known, in light of this knowledge prior opinion on μ_P_ and δ would follow a (2-dimensional) Gaussian distribution with mean vector (μ_P0_, δ_0_) and variance-covariance matrix (1/τ)R. The prior means μ_P0_ and δ_0_ represent what consensus best guesses at μ_P_ and δ would be in light of τ, while the variance-covariance matrix quantifies uncertainty about these guesses and the correlation of opinion on the two parameters.

Given an expert’s prior opinion on μ_P_, δ and τ, one can derive their predictive distribution for the response of a future patient randomised to either pamidronate or adalimumab. An expert’s uncertainty about how a future individual patient will respond reflects uncertainty arising due to sampling variability (i.e., two patients given the same treatment will respond differently) and the expert’s imperfect prior understanding of the data generating parameters. Further details on the proposed statistical model for data from a future CRMO trial, and expert prior opinion on the three key statistical parameters, can be found in S1 Appendix.

### Establishing expert opinion

It would be a challenging and complex task to elicit expert opinion directly on μ_P_, δ and τ. Instead we took a different approach, characterising opinion on these quantities by asking experts a sequence of ten questions to establish their opinion on the change in pain score that would be observed if a new patient were treated with either pamidronate or adalimumab for 26 weeks according to the proposed trial protocol. Five questions concerned a patient’s response after treatment with pamidronate; five concerned adalimumab. The 10 questions were initially drafted by a statistician (LVH) and were then critically reviewed by clinical members of the team (AR, MWB) to check for clarity of language and meaning. The complete questionnaire is provided in S1 Appendix. To make elicitation questions more concrete, experts were asked to consider a typical patient who presents at baseline with a pain score of 60 mm, and to give their opinion on the pain score that this patient would record after 26 weeks of treatment. From this, opinion on the change from baseline could be derived. We then deduced the prior distributions for μ_P_, δ and τ that would be most consistent with the expert’s stated opinions on how a future patient would respond. A bespoke web application, written in R [28] using the Shiny package [29], was developed to facilitate the elicitation process. It takes an expert’s answers to the ten elicitation questions and feeds back graphical and descriptive summaries of fitted prior distributions. The software is freely available from the authors upon request.

To obtain a more complete understanding of each expert’s opinions, they were also asked to complete a table, assigning to a number of intervals weights representing the strength of their belief that a new typical patient’s 26-week pain score on pamidronate would lie in that interval. A similar table was completed for adalimumab. The tables which appear in questions P6 and A6 were adapted from those used by White et al [30] in their elicitation exercise for the CHARM trials (although the parameters of interest in that example were quite different to those of interest for the CRMO trial). This process also enabled statistical facilitators to verify the consistency of an expert’s answers to earlier elicitation questions.

#### Establishing consensus expert opinion

To deduce prior distributions for μ_P_, δ and τ which reflected the group’s consensus position, we adopted the following dynamic strategy. We used as a starting point the priors implied by the arithmetic means of the experts’ final individual answers to the 10 key elicitation questions, and fedback to the group visualisations and summaries of these distributions to confirm their acceptability. If they were deemed to be unsuitable as consensus priors, our next step would be to identify why and explore a range of alternative distributions created by modifying the mean elicitation answers appropriately. This approach seemed reasonable in light of our experiences with the MYPAN prior elicitation exercise [23]. Furthermore, taking the mean of the individual elicitation answers is a natural starting point when there is little variation between the experts’ stated individual opinions. However, when defining consensus priors for each parameter, we were also prepared to respond dynamically to the group discussions. For example, if it became clear that a consensus position would not be possible or that our statistical model for expert opinions was fitting poorly, we would document the expert’s individual answers to the elicitation questions and the group discussion, and then consider next steps off-line.

### Evaluating the operating characteristics of a future CNO/CRMO trial

To explore the impact that data from a future CNO/CRMO trial randomising 20 patients to each treatment arm would have on expert opinion, we investigated how the consensus priors would be updated by three hypothetical trial datasets. Each hypothetical dataset was summarised by its corresponding estimates of mean changes from baseline on adalimumab and pamidronate (${\bar{x}}_{A}$ and ${\bar{x}}_{P}$, respectively), and the pooled outcome variance estimate (s^2^). These summaries are ‘complete’ in the sense that any two trials with individual patient data leading to the same values of these estimates would produce the same posterior distributions. The three hypothetical datasets were specified after the consensus priors had been formally agreed upon at the elicitation meeting. One dataset was stipulated so as to be consistent with the consensus priors. The remaining datasets were designed to conflict with the consensus prior opinion.

To quantify the operating characteristics of a CNO/CRMO trial recruiting 20 patients to each treatment arm, we performed a simulation study. Data were simulated under the six scenarios listed in Table 2, assuming patient outcomes followed the normal linear model described in Section ‘Defining the quantities to be elicited’. We considered three scenarios for the average change from baseline pain score at 26 weeks on pamidronate:

- μ_P_ is equal to the mode of its consensus prior
- μ_P_ = -40mm (i.e. 24% smaller than its prior mode; pamidronate more effective than expected)
- μ_P_ = -26mm (i.e. 20% larger than its prior mode; pamidronate less effective than expected).

**Table 2:** Data simulation scenarios. Data stimulation scenarios in which: μ_P_ (or μ_A_) denotes the true long-run average change from baseline pain score at 26 weeks on pamidronate (or adalimumab); σ is the common standard deviation of change from baseline pain scores.

True average change from baseline on pamidronate	True difference between average changes from baseline at 26 weeks on pamidronate and adalimumab	
	No difference	Pamidronate superior by clinically relevant margin
Scenario A	μ_P_ = -32.3, μ_A_ = -32.3, σ = 9.3	μ_P_ = -32.3, μ_A_ = -24.9, σ = 9.3
Scenario B	μ_P_ = -40, μ_A_ = -40, σ = 11.5	μ_P_ = -40, μ_A_ = -30.8, σ = 11.5
Scenario C	μ_P_ = -26, μ_A_ = -26, σ = 7.5	μ_P_ = -26, μ_A_ = -20, σ = 7.5

These scenarios were chosen to capture a range of values for μ_P_ which looked plausible in light of the experts’ consensus prior opinion and which rounded to whole numbers (for convenience).

In scenarios A-C, responses on adalimumab were simulated under two different cases for the treatment effect: i) no treatment effect, in which case the average change from baseline on adalimumab is equal to μ_P_; ii) a clinically relevant treatment effect, in which case the average change from baseline on pamidronate is 30% smaller than the average change on adalimumab. On completion of each simulated trial we calculated the posterior probability that either pamidronate is beneficial on average and superior to adalimumab by the clinically relevant margin defined above; or adalimumab is beneficial and superior to pamidronate by a clinically relevant margin. In each of the six simulation scenarios, we recorded the proportion of 1,000 simulated trials in which this posterior probability exceeded 0.2. All simulations were run in R using the OpenBUGS [31] software to implement the Bayesian analysis.

## Results

Fifty-one clinicians from across Europe, Turkey and Russia who completed the electronic survey, registered their interest in attending the CNO/CRMO consensus meeting, 32 of whom met predefined “expert criteria”. Three experts were based in Turkey or Russia, and due to funding constraints were not considered further for participation in the meeting. Fifteen of the remaining eligible experts, representing eleven clinical centres, were based in the UK (fourteen experts) or Ireland (one expert). These experts were first listed in alphabetical order; in cases where more than one expert had the same affiliation, only the expert occurring highest in the alphabetical ordering was retained. From the reduced listing of eleven experts thus compiled, six were randomly selected (BJ, JD, AK, OGK, SCL, MR): three of these experts represented a centre that had more than one volunteer. In these cases, all of the experts from that centre were contacted and invited to nominate a single representative to attend the consensus meeting.

The remaining 14 eligible experts from the rest of Europe were based in nine countries, namely, Denmark (2 experts), France (1), Germany (5), Greece (1), Italy (1), Netherlands (1), Spain (1), Sweden (1) and Switzerland (1). From these countries, we randomly selected four (Denmark, Germany, Greece, Netherlands) and then one expert per selected country (TH, JS, DV, CH). In addition to the two key opinion leaders invited from North America, shortly prior to the consensus meeting, funding became available to cover the travel expenses of an additional UK-based expert (MR). Therefore, in total twelve experts attended the meeting, all of whom are included as co-authors on this manuscript.

## Consensus opinion

Table 3 lists answers of the thirteen experts to the ten principal elicitation questions (labelled QP1-5 and QA1-5). The group accepted as their consensus answers to these questions the arithmetic means of their individual answers. Consensus opinion was that a typical new patient treated with pamidronate would have an 84% (chance of registering some improvement in his/her pain score relative to baseline at 26 weeks and an 83% chance when treated with adalimumab. Experts thought there was a 50% chance that a new typical patient on pamidronate would record a pain score of 28 mm or better at 26 weeks and a pain score of 30 mm on adalimumab. These answers reflect the general opinion that a typical patient’s 26-week pain score would be broadly similar after treatment with pamidronate or adalimumab.

**Table 3:** Expert responses to elicitation questions.

Expert	Pamidronate					Adalimumab				
	QP1	QP2	QP3	QP4	QP5	QA1	QA2	QA3	QA4	QA5
1	75	50	40	5	0	80	40	35	5	0
2	85	25	15	10	5	80	30	25	20	15
3	80	40	30	20	10	70	50	40	30	20
4	90	40	30	20	10	85	45	35	25	15
5	90	35	25	20	15	75	45	35	25	20
6	95	40	20	5	2	75	60	40	15	5
7	85	40	30	20	10	75	50	40	30	10
8	90	50	40	30	20	95	50	35	20	10
9	90	40	30	20	10	95	35	25	15	5
10	90	65	30	20	10	95	60	30	20	10
11	80	50	20	5	0	80	60	30	20	4
12	85	40	20	0	0	95	20	10	0	0
13	60	40	30	25	15	80	15	10	5	0
**Mean**	84.2	42.7	27.7	15.4	8.2	83.1	43.1	30	17.7	8.8
**Median**	85	40	30	20	10	80	45	35	20	10

Experts’ final answers to ten structured elicitation questions asking for opinion on the pain score of a typical patient after 26 weeks of treatment with either pamidronate or adalimumab according to the proposed CRMO protocol. The wording of the elicitation questions is listed in S1 Appendix. The arithmetic means of answers were proposed and accepted by the group as summaries of its consensus opinion.

Prior distributions for: a) the response of a future individual patient; b) the average change in pain score on pamidronate; and c) the difference between average changes in pain score on adalimumab and pamidronate, corresponding to these ten consensus answers were then derived, and visualisations and summaries of the priors fed back to the group. Experts agreed to adopt these proposals as the group’s consensus prior distributions.

**Fig 1:**
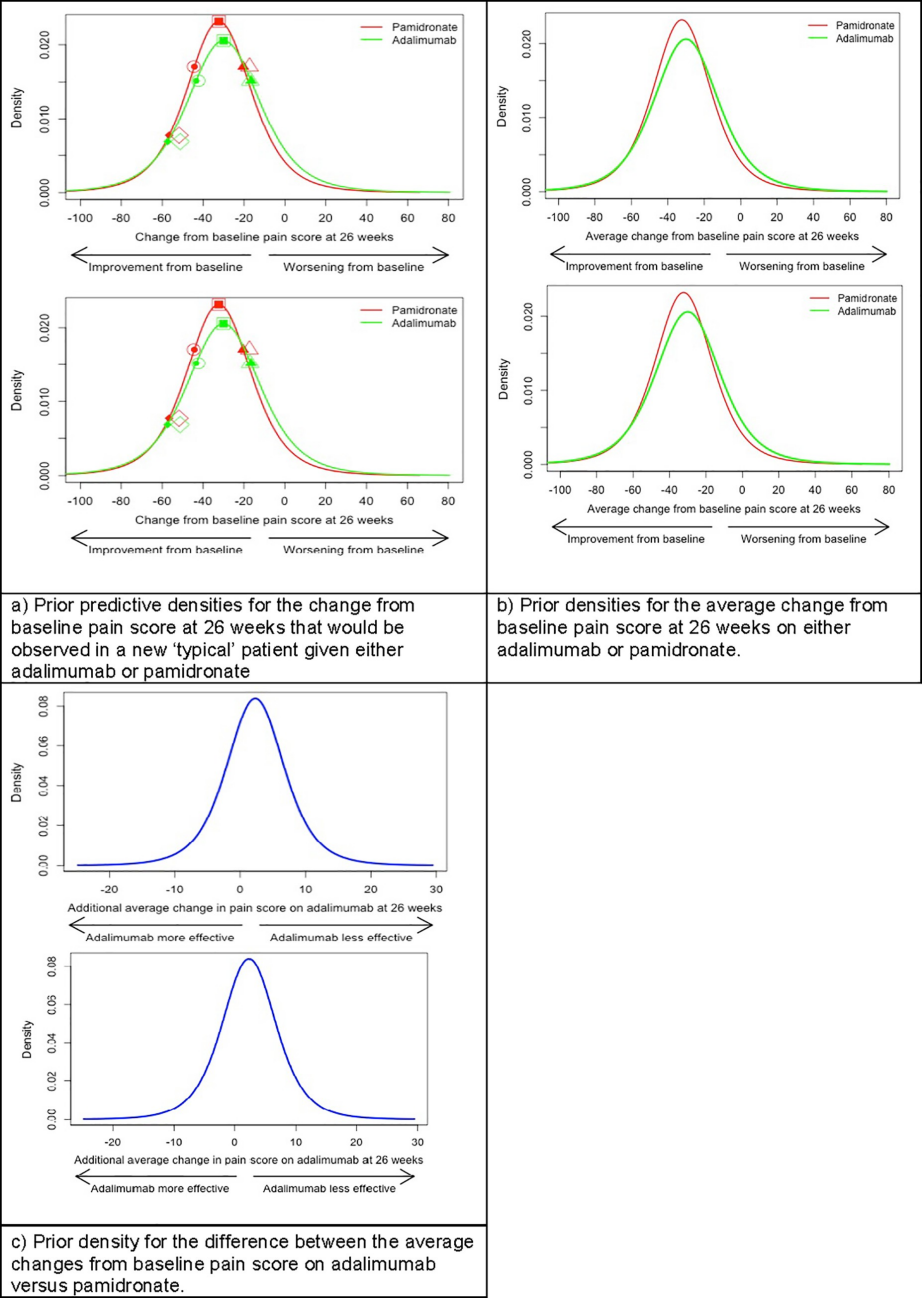
Consensus prior densities for outcome model parameters. (Fig 1B) shows consensus prior distributions for the average change in pain scores at 26 weeks on pamidronate or adalimumab. Prior modal values for the average change in pain score on pamidronate and adalimumab are -32.3 mm and -30 mm, respectively. (Fig 1C) displays the consensus prior for the treatment effect. Prior opinion is that there is a 90% chance that the true difference between average change scores on adalimumab versus pamidronate lies between -6.9 mm and 11.5 mm (with a negative difference indicating an advantage for adalimumab). Furthermore, there is a 68.4% prior probability that pamidronate is superior to adalimumab by any margin. However, it is clear that there is considerable uncertainty about the precise relative merits of the two treatments. (Fig 1A), plotted symbols represent the experts’ consensus answers to elicitation questions QA2-5 and QP2-5 minus 60mm, which was the pain score assumed to characterise a typical patient at baseline.

Fitted prior distributions corresponding to consensus answers are shown in (Fig 1). (Fig 1A) plots fitted predictive distributions for a new typical patient’s change in pain score at 26 weeks. Due to the pragmatic nature of the Bayesian outcome model, fitted predictive distributions place prior weight on changes ranging between +/- 100mm, but place negligible weight on values outside this range. Percentiles of the fitted prior predictive distributions for a new typical patient’s change in pain score at 26 weeks (shown as solid plotting symbols) are close to the experts’ stated beliefs (indicated by open symbols) for both treatments.

## Impact of consensus opinion on planned clinical trial

Three hypothetical datasets from a future CRMO trial randomising 40 patients between pamidronate and adalimumab were defined as follows:

- Hypothetical dataset 1: s^2^ = 4.6, ${\bar{x}}_{A}$ = -30, ${\bar{x}}_{P}$ = -30. These estimates are broadly consistent with consensus prior opinion.
- Hypothetical dataset 2: s^2^ = 21.3, ${\bar{x}}_{A}$ = -30, ${\bar{x}}_{P}$ = -20. The variance estimate is larger than would be expected from prior opinion; the estimated mean outcome on pamidronate is disappointing; adalimumab is more effective than pamidronate.
- Hypothetical dataset 3: s^2^ = 4.6, ${\bar{x}}_{A}$ = -10, ${\bar{x}}_{P}$ = -20. The variance estimate is consistent with prior opinion, while the estimated mean outcomes on both treatments are disappointing, although pamidronate is more effective than adalimumab.

**Fig 2:**
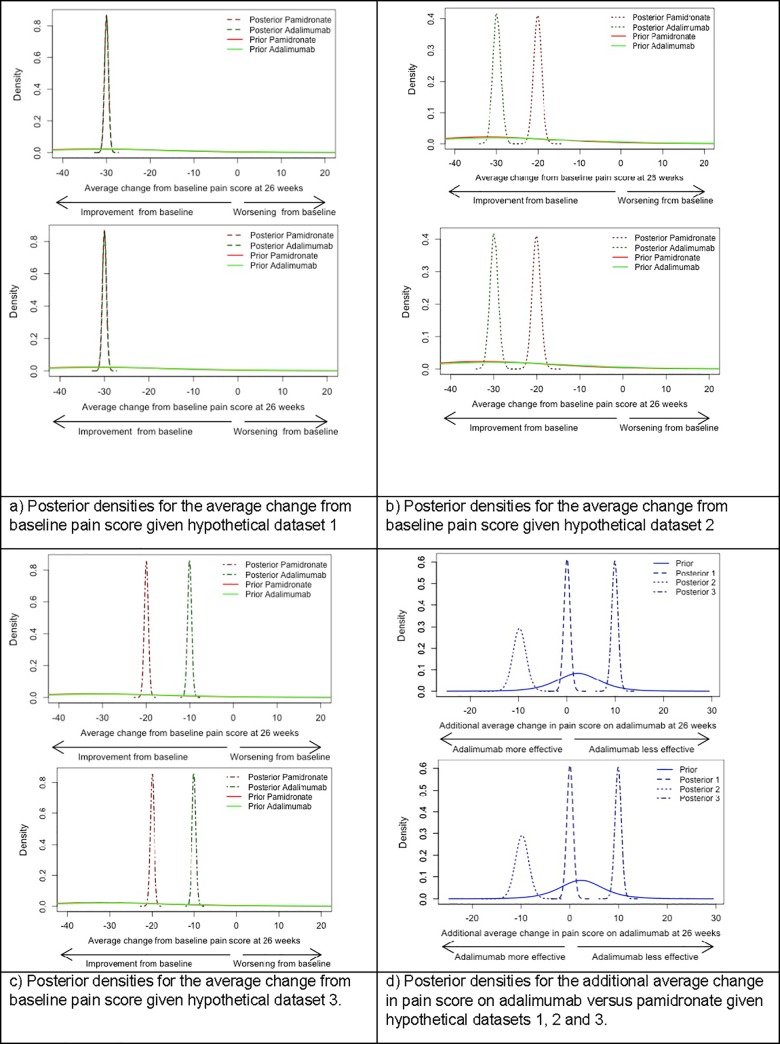
Posterior densities for model parameters.

When interpreting the outcome variance estimate, we note that 4.6 mm is the 75^th^ percentile of the consensus prior distribution for the outcome variance, while √(21.3) is twice the prior modal estimate of the treatment difference.

(Fig 2A–2D) illustrate how consensus prior opinion on the true average changes in pain score on each treatment, and their difference, would be updated by each hypothetical dataset. Comparing prior and posterior densities, it is clear that data on 40 patients would have an important impact on the current consensus understanding of the effects of pamidronate and adalimumab in CNO/CRMO patients. Posterior densities become more peaked, attributing plausibility to a smaller range of parameter values. In addition, data on 40 patients will be able to shift prior opinion in the event that they are inconsistent with beliefs represented by the consensus priors.

Posterior densities for model parameters that would be obtained by updating consensus prior distributions with hypothetical data on 40 patients.

The posterior 90% credible interval for the average change from baseline pain score at 26 weeks on adalimumab was (-30.8, -29.2) mm given hypothetical dataset 1; (-31.5, -28.3) mm given hypothetical dataset 2; and (-10.8, -9.3) mm given hypothetical dataset 3.

The posterior 90% credible interval for the average change from baseline pain score at 26 weeks on pamidronate was (-30.8, -29.3) mm given hypothetical dataset 1; (-21.7, -18.5) mm given hypothetical dataset 2; and (-20.7, -19.2) mm given hypothetical dataset 3.

The posterior 90% credible interval for the additional average change in pain score experienced on adalimumab is (-1.0, 1.1) mm given hypothetical dataset 1; (-12.1, -7.5) mm given hypothetical dataset 2; (8.8, 11.0) mm given hypothetical dataset 3.

The results of the simulation study in Table 4 show that the proposed Bayesian decision rule would control the risk of incorrectly declaring a clinically relevant difference between treatments when none exists at an acceptable level. In simulation scenario A, when there is a clinically important difference between pamidronate and adalimumab, 76% of simulated trials correctly declared a difference according to the proposed Bayesian decision rule. Similar probabilities were also recorded in scenarios B and C.

**Table 4:** Simulated trials.

True long-run average response on pamidronate	True difference between average changes from baseline at 26 weeks	
	No difference	Pamidronate superior by clinically relevant margin
Scenario A	0.045	0.76
Scenario B	0.04	0.776
Scenario C	0.036	0.778

Proportion of 1,000 simulated trials which concluded that there was evidence of a clinically relevant difference in any direction between pamidronate and adalimumab. All simulated trials allocated 20 patients to each treatment arm.

## Discussion

There are no published RCTs comparing the relative effectiveness of adalimumab and pamidronate in treating children with CNO/CRMO [8, 16]. The rarity of CNO/CRMO leads to significant challenges in conducting RCTs with sufficient power to provide a definitive outcome. We proposed this using a Bayesian clinical trial design and have described the process and outcome of the elicitation exercise to establish expert prior opinion. The process was done in a structured format [27], led by a statistician experienced in Bayesian statistics (LVH), and informed by a systematic review [32] providing principles for best practice in prior opinion elicitation.

Importantly, the priors’ elicitation meeting with international experts in the field concluded that a Bayesian RCT with data on 40 patients would have an important impact on the current consensus understanding of the effects of pamidronate and adalimumab in CNO/CRMO patients. Specifically, these data will be able to shift prior opinion in the event that they are inconsistent with beliefs represented by the consensus priors. Furthermore, the proposed Bayesian decision rule will control the risk of incorrectly declaring a clinically relevant difference between treatments when none exists at an acceptable level.

The results of the experts Bayesian priors clearly demonstrated that there was consensus on the equivocal efficacy of both agents considered in planned trial. There was significant agreement that a trial as planned was required to help manage children and adolescents with this condition. It is important to reflect that most of the experts manage children with CRMO on a regular basis and understand the existing lack of clarity on appropriate treatment modalities for these children. It is interesting to note that consensus treatment plans (CTPs) published by the CARRA (Childhood Arthritis and Rheumatology Research Alliance) group demonstrate similar therapeutic dilemma with both agents considered in our trial being considered as equally effective based on limited existing evidence [33].

This exercise will now also allow us to embark on our planned study with priors informing us of the need for this study and reaffirming the planned study design in light of existing evidence base.

We acknowledge that inviting experts to volunteer in the process may impact on the type of expert participating, possibly more likely to attract those more supportive of the trial or those more critical of it. However, although using the face-to-face elicitation process may limit the numbers of experts’ opinions that can be included; we felt it is important to ensure that the process is highly interactive so that the process is effective in capturing the true opinion of the experts as well as allowing structured group discussions. We have attempted to overcome this through inviting experts over a wide range of locations using a clear and transparent expert selection process. One limitation of the prior elicitation meeting is that we did not record ahead of time the potential conflicts of interest (CoIs) of the experts who participated, and so we are unable to comment on the potential impact these may have had on the opinions expressed. The SHELF elicitation framework [34] recommends that experts record ahead of the meeting any personal interests in the outcome of the elicitation exercise: the aim is not to exclude experts with conflicts but rather to be completely transparent about them.

In conclusion, we have defined consensus prior opinion regarding the relative effectiveness of adalimumab and pamidronate in reducing pain scores in children with CNO/CRMO. This opinion will be tested in the planned CNO/CRMO clinical trial of adalimumab and pamidronate. Demonstration of the effectiveness of the process for establishing expert consensus opinion in rare diseases will be helpful for consideration of developing clinical trials in other rare, paediatric diseases.

## Supporting information

S1 Appendix Questionnaire to elicit individual experts’ prior opinions. (DOCX)
