# Supplementary material for: Defining consensus opinion to develop randomised controlled trials in rare diseases using Bayesian design: An example of a proposed trial of adalimumab versus pamidronate for children with CNO/CRMO
Source: PLoS One. 2019 Jun 5;14(6):e0215739. doi: 10.1371/journal.pone.0215739 (PMC6550371; doi:10.1371/journal.pone.0215739)
Supplement: S1 Appendix — (DOCX) [file pone.0215739.s001.docx]

**Appendix A: Questionnaire to elicit individual experts’ prior opinions**

**Introduction:** The questions below assume that a patient’s pain will be measured on a 100mm visual analogue scale (VAS). Higher scores indicate greater pain, such that:

- a score of 0 indicates the complete absence of pain;
- a score of 100 indicates unbearable pain.

**Eliciting your opinion on the effect of pamidronate**

Suppose you are asked to treat an individual patient. Let’s suppose that this patient is typical of the patients you see in your clinical practice and **presents at baseline with a pain score of 60mm on the VAS**. The following questions ask for your opinion on what this patient’s pain score would be after 26 weeks of treatment with pamidronate.

1. Provide a value (between 0 and 100%) which you think is the percentage chance that the patient would have a final pain score better than 60 after 26 weeks treatment with pamidronate.

Your answer: __________________________________

**Example: The more confident you are that the patient’s pain would improve (by even the slightest margin) over the 26 weeks, the closer to 100% your answer should be.**

1. Provide a number between 0 and 100 such that you are 75% sure that the patient’s final pain score at 26 weeks would be better than this value.

Your answer: __________________________________

**Example 1: An answer of 50 reflects the opinion that there is a 75% chance the patient’s pain score at 26 weeks would be 50 or better. This states that there is a large chance the patient will have improved relative to baseline.**

**Example 2: An answer of 70 reflects the opinion that there is a 75% chance the patient’s pain score at 26 weeks would be 70 or better. This admits a larger chance that the patient’s pain score may have deteriorated relative to baseline by 26 weeks.**

1. Provide a number between 0 and 100 such that you are 50% sure that the patient’s final pain score at 26 weeks would be better than this value.

Your answer: __________________________________

**Example: An answer of 40 reflects the opinion that there is a 50% chance the patient’s pain score at 26 weeks would be 40 or better (and a 50% chance the patient’s pain score would be worse than 40).**

1. Provide a number between 0 and 100 such that you are 25% sure that the patient’s final pain score at 26 weeks would be better than this value.

Your answer: __________________________________

**Example: An answer of 32 reflects the opinion that there is a 25% chance the patient’s pain score at 26 weeks would be 32 or better (and a 75% chance the patient’s pain score would be worse than 32).**

1. Provide a number between 0 and 100 such that you are 10% sure that the patient’s final pain score at 26 weeks would be better than this value.

Your answer: __________________________________

**Example: An answer of 20 reflects the opinion that there is a 10% chance the patient’s pain score at 26 weeks would be 20 or better (and a 90% chance the patient’s pain score would be worse than 20).**

1. Consider a typical patient presenting at baseline with a pain score of 60mm who will receive pamidronate. Enter in the table below the percentage chance (between 0 – 100) that represents the strength of your belief that this patient would actually have a pain score at 26 weeks lying in each of the intervals provided. The more strongly you believe that the patient’s score would actually lie in a particular interval, the closer to 100 your number should be. If you believe it impossible that the score would lie in a particular interval, then the number should be zero. If you are certain that the score would lie in an interval, the number should be 100. Your scores should all add up to 100.

|  | Improvement in pain at 26 weeks compared with baseline | | | | | | | Worsening of pain at 26 weeks compared with baseline | | | | |
| --- | --- | --- | --- | --- | --- | --- | --- | --- | --- | --- | --- | --- |
| Final pain score at 26 weeks | < 5 | 5 -10 | 10-20 | 20-30 | 30-40 | 40-50 | 50-60 | 60-70 | 70-80 | 80-90 | 90-95 | >95 |
| Your entry |  |  |  |  |  |  |  |  |  |  |  |  |
| **Example** | **0** | **0** | **0** | **0** | **40** | **40** | **20** | **0** | **0** | **0** | **0** | **0** |

This example reflects a strong belief that the patient will see a marked improvement in their pain after 26 weeks. For example, there is an equal chance of the patient’s final pain score being between 40 - 50 (representing a 17 - 33% improvement from an initial score of 60) or 30 – 40 (representing a 33 - 50% improvement from baseline). There is also a smaller chance that the patient experiences a more modest improvement in their pain score (with their final score lying between 50 and 60). All other final scores are considered impossible and therefore entered as zero.

**Eliciting your opinion on the effect of adalimumab**

Suppose you are asked to treat an individual patient. Let’s suppose that this patient is typical of the patients you see in your clinical practice and **presents at baseline with a pain score of 60mm on the VAS**. The following questions ask for your opinion on what this patient’s pain score would be after 26 weeks of treatment with adalimumab.

1. Provide a value (between 0 and 100%) which you think is the percentage chance that the patient would have a final pain score better than 60 after 26 weeks treatment with adalimumab.

Your answer: __________________________________

**Example: The more confident you are that the patient’s pain would improve (by even the slightest margin) over the 26 weeks, the closer to 100% your answer should be.**

1. Provide a number between 0 and 100 such that you are 75% sure that the patient’s final pain score at 26 weeks would be better than this value.

Your answer: __________________________________

**Example 1: An answer of 50 reflects the opinion that there is a 75% chance the patient’s pain score at 26 weeks would be 50 or better. This states that there is a large chance the patient will have improved relative to baseline.**

**Example 2: An answer of 70 reflects the opinion that there is a 75% chance the patient’s pain score at 26 weeks would be 70 or better. This admits a larger chance that the patient’s pain score may have deteriorated relative to baseline by 26 weeks.**

1. Provide a number between 0 and 100 such that you are 50% sure that the patient’s final pain score at 26 weeks would be better than this value.

Your answer: __________________________________

**Example: An answer of 40 reflects the opinion that there is a 50% chance the patient’s pain score at 26 weeks would be 40 or better (and a 50% chance the patient’s pain score would be worse than 40).**

1. Provide a number between 0 and 100 such that you are 25% sure that the patient’s final pain score at 26 weeks would be better than this value.

Your answer: __________________________________

**Example: An answer of 32 reflects the opinion that there is a 25% chance the patient’s pain score at 26 weeks would be 32 or better (and a 75% chance the patient’s pain score would be worse than 32).**

1. Provide a number between 0 and 100 such that you are 10% sure that the patient’s final pain score at 26 weeks would be better than this value.

Your answer: __________________________________

**Example: An answer of 20 reflects the opinion that there is a 10% chance the patient’s pain score at 26 weeks would be 20 or better (and a 90% chance the patient’s pain score would be worse than 20).**

1. Consider a typical patient presenting at baseline with a pain score of 60mm who will receive adalimumab. Enter in the table below the percentage chance (between 0 – 100) that represents the strength of your belief that this patient would actually have a pain score at 26 weeks lying in each of the intervals provided. The more strongly you believe that the patient’s score would actually lie in a particular interval, the closer to 100 your number should be. If you believe it impossible that the score would lie in a particular interval, then the number should be zero. If you are certain that the score would lie in an interval, the number should be 100. Your scores should all add up to 100.

|  | Improvement in pain at 26 weeks compared with baseline | | | | | | | Worsening of pain at 26 weeks compared with baseline | | | | |
| --- | --- | --- | --- | --- | --- | --- | --- | --- | --- | --- | --- | --- |
| Final pain score at 26 weeks | < 5 | 5 -10 | 10-20 | 20-30 | 30-40 | 40-50 | 50-60 | 60-70 | 70-80 | 80-90 | 90-95 | >95 |
| Your entry |  |  |  |  |  |  |  |  |  |  |  |  |
| **Example** | **0** | **0** | **0** | **0** | **40** | **40** | **20** | **0** | **0** | **0** | **0** | **0** |

**Appendix B: Statistical details of the Bayesian procedure**

**B.1 Bayesian model for the primary outcome: Change from baseline in pain score at 26 weeks**

Suppose that n patients are to be recruited into the CRMO trial, with n_A_ receiving treatment A (adalimumab) and n_P_ receiving treatment P (pamidronate). Letting Y_i_ denote the primary response of patient i randomised to receive treatment t_i_, we model

Y_i_ = μ_P_ + βt_i_ + ε_i_, (1)

where ε_i_ ~ N(0, σ^2^), for i = 1, .., n, are independent random errors and t_i_ is an indicator variable taking the value 1 if patient i is randomised to A, and 0 otherwise. Under this model, μ_P_ represents the expected change in pain score on P, while β can be interpreted as a measure of difference between A and P. Since negative changes imply an improvement in patient health, β will be negative if A is superior to P. Defining μ_A_ as the expected response on A, we can write β = μ_A_ – μ_P_. Furthermore, let τ = σ^-2^.

Prior opinion on the parameters of model (1) will be modelled using conjugate distributions, that is,

$\boldsymbol{\eta|}\tau\sim N(\boldsymbol{\eta}_{0}, \tau^{-1}R)$ and $\tau\sim\text{Ga}(a_{0}, b_{0})$, (2)

which implies that conditional prior opinion on **η** = (μ_P_, β)^T^ given τ follows a bivariate normal distribution with mode **η**_0_ = (η_00_, η_01_)^T^ and variance proportional to R, where

$$R= \left( \begin{matrix} r_{11} & r_{12} \\ r_{12} & r_{22} \end{matrix} \right)$$

is a (2 x 2) symmetric positive-definite matrix. Thus, model (2) can accommodate correlated prior opinion on μ_P_ and β. Meanwhile, prior opinion on τ is expressed as a Gamma distribution with shape and rate parameters a_0_ and b_0_. Under this Bayesian model, the marginal prior distribution for $\boldsymbol{\eta}$ follows a bivariate Student t-distribution St_2_($\boldsymbol{\eta}_{0}$, a_0_R^-1^/b_0_, 2a_0_), where St_k_(**μ**, **λ**, ρ) denotes a k-dimensional Student t-distribution with expectation **μ** and variance matrix **λ**^-1^(ρ/(ρ-2)) (p.139, Bernardo JM & Smith AFM, *Bayesian Theory*, 2000, Wiley).

We can characterise the prior opinion on **η** and τ by considering the predictive distributions for observations on P and A that model (2) implies. Let X_P_ represent the response of a new patient randomised to P, and let X_A_ represent the response of a new patient randomised to A. Furthermore, let St(δ, φ, ρ) denote a Student t distribution with mode and expectation δ; degrees of freedom ρ; and variance ρ/[(ρ - 2)φ]. Then, given model (2), it follows that X_P_ and X_A_ have prior predictive distributions X_P_ ~ St(η_00_, a_0_[b_0_r_11_(r_11_^-1^ + 1)]^-1^, 2a_0_) and X_A_ ~ St(η_00_ + η_01_, a_0_[zb_0_ (z^-1^ + 1)]^-1^, 2a_0_), where z = r_11_ + r_22_ + 2r_12_.

**B.2 Deriving posterior distributions for model parameters**

We collect data **Y**|$\boldsymbol{\eta}$**,** τ ~ N(t$\boldsymbol{\eta}$**,** $\tau^{-1}$I_nxn_), where t is an (n x 2) design matrix consistent with model (1) and the scenario that n/2 patients are randomised to treatment A and n/2 are randomised to treatment P. Suppose we observe **Y = y**. Then, posterior distributions for the model parameters are:

$\boldsymbol{\eta}$**|y**, τ ~ N( (t^T^t + R^-1^)^-1^(t^T^**y** + R^-1^ $\boldsymbol{\eta}$_0_**),** $\tau^{-1}$(t^T^t + R^-1^)^-1^)

τ|**y** ~ Gamma(n/2 + a_0_, b_0_ + 0.5*(**y**^T^**y** + $\boldsymbol{\eta}_{0}^{T}$R^-1^$\boldsymbol{\eta}$_0_ – (t^T^**y** + R^-1^$\boldsymbol{\eta}$_0_)^T^(t^T^t + R^-1^)^-1^(t^T^**y** + R^-1^$\boldsymbol{\eta}$_0_))) and

$\boldsymbol{\eta}$**|y** ~ St_2_(**(t^T^t + R^-1^)^-1^(t^T^y + R^-1^**$\boldsymbol{\eta}$_0_), $\Sigma_{p}^{-1}$, n + 2a_0_), where

$\Sigma_{p}$= [2(t^T^t + R^-1^)^-1^/(n + 2a_0_)][b_0_ + 0.5(**y**^T^**y** + $\boldsymbol{\eta}_{0}^{T}$R^-1^$\boldsymbol{\eta}$_0_ – (t^T^**y** + R^-1^$\boldsymbol{\eta}$_0_)^T^(t^T^t + R^-1^)^-1^(t^T^**y** + R^-1^$\boldsymbol{\eta}$_0_))].
